# Assessing the possibilities of designing a unified multistep continuous flow synthesis platform

**DOI:** 10.3762/bjoc.14.166

**Published:** 2018-07-26

**Authors:** Mrityunjay K Sharma, Roopashri B Acharya, Chinmay A Shukla, Amol A Kulkarni

**Affiliations:** 1 Academy of Scientific and Innovative Research (AcSIR), CSIR-National Chemical Laboratory (NCL) Campus, Pune 411008, India; 2 Chem. Eng. & Proc. Dev. Div., CSIR-National Chemical Laboratory, Dr. Homi Bhaba Road, Pashan, Pune 411008, India

**Keywords:** automation, continuous flow synthesis, cybernetics, multistep flow synthesis, unified platforms

## Abstract

The multistep flow synthesis of complex molecules has gained momentum over the last few years. A wide range of reaction types and conditions have been integrated seamlessly on a single platform including in-line separation as well as monitoring. Beyond merely getting considered as ‘flow version’ of conventional ‘one-pot synthesis’, multistep flow synthesis has become the next generation tool for creating libraries of new molecules. Here we give a more ‘engineering’ look at the possibility of developing a ‘unified multistep flow synthesis platform’. A detailed analysis of various scenarios is presented considering 4 different classes of drugs already reported in the literature. The possible complexities that an automated and controlled platform needs to handle are also discussed in detail. Three different design approaches are proposed: (i) one molecule at a time, (ii) many molecules at a time and (iii) cybernetic approach. Each approach would lead to the effortless integration of different synthesis stages and also at different synthesis scales. While one may expect such a platform to operate like a ‘driverless car’ or a ‘robo chemist’ or a ‘transformer’, in reality, such an envisaged system would be much more complex than these examples.

## Review

### Introduction

Flow chemistry is now seen as a reliable approach for the synthesis of simple organic compounds [1–6], complex large molecular weight medicinal drugs [7–12], polymeric materials [13–15], nanomaterials (metallic, bimetallic, composites, metal oxides, etc.) [16–18], catalysts [7,19], etc. In the recent times, the applicability of this tool has been extended for the synthesis of high value drugs involving multiple reaction steps including separation protocols [8–9,20]. A vast range of useful molecules that are synthesized in flow has also helped integrate the complex synthesis with fine engineering to make the systems completely automated [9,20]. Flow chemistry gains its benefits from excellent heat and mass transfer rates and rapid mixing which is not possible in the case of conventional synthesis modes [21]. In general, the continuous flow synthesis aims at conducting the reactions at intrinsic kinetics. This helps to have reactors having smaller volumes making them inherently safer. Due to low processing volumes and reactions at intrinsic rates without much of human intervention it is possible to carry out hazardous reactions and a reaction at much higher temperature which is not possible with conventional methods [22–23]. An automated flow synthesis approach also reduces the labor costs significantly and operation can go on for a long time without any interruptions or significant downtime for the maintenance [9,20]. Many reactions have been performed in flow synthesis and are shown to be better than conventional synthesis [24–27]. A few examples of experimental set-ups of successfully demonstrated multistep flow synthesis encompassing various kinds of reactions from the literature are given in Table 1.

**Table 1:** Reactions and corresponding flow synthesis set-up from the literature.

reaction name and flow set-up

**Grignard reaction** [28] 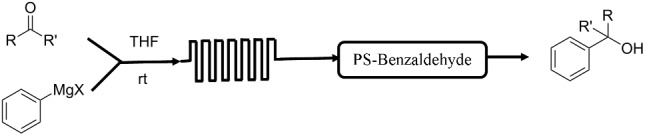

**Curtius rearrangement** [29] 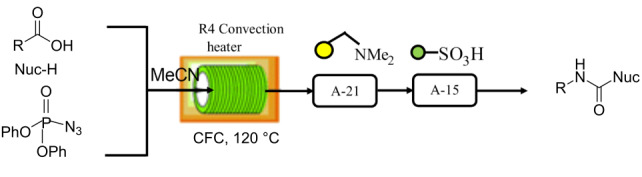

**Heck reaction** [30] 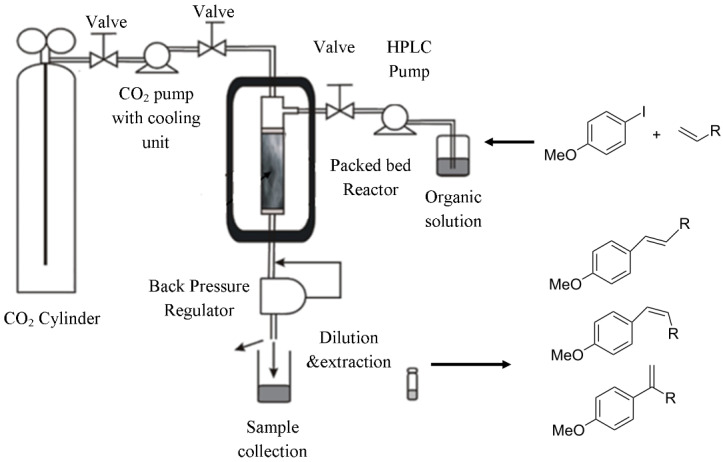
**Cannizzaro oxidation reaction** [31] 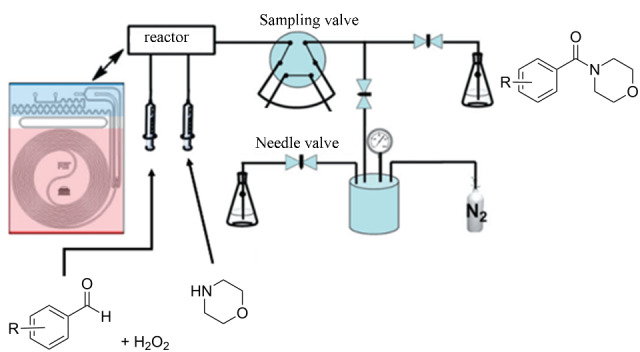

**Biginelli reaction** [32] 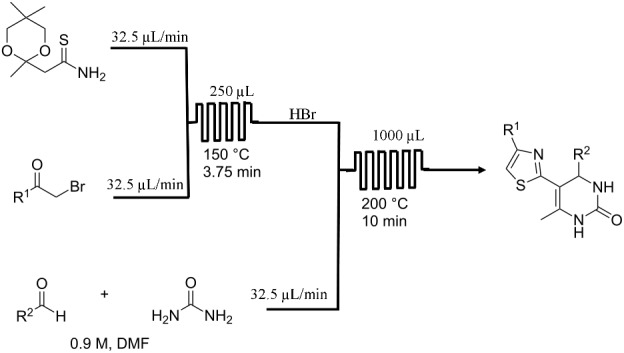

Single step approaches were useful in terms of evaluating the concepts in continuous flow synthesis. However, since synthesis of any fine chemical or medicinal drug or agrochemical compound involves a sequence of reactions as well as several unit operations, by making only one process step continuous does not make much impact in terms of overall efficiency, economics and operation time. Thus the flow synthesis made its mark in terms of improving the product quality and reducing the environmental impact, albeit only for single reactions. This also helped to understand the safety related issues of flow synthesis and even helped to study the effect of operating parameters (viz. flow rates, temperature, pressure, pH, etc.) and design parameters (viz. mixing, heat transfer, mass transfer, dispersion, etc.), which together helped in developing reactor selection protocols and safer intensification window for its continuous operation. Over the time even the process control structures also got evolved for specific kind of experimental set-ups and even automated self-optimizing platforms were also tested [33]. The natural evolution was an archetype for the multistep flow synthesis. The integration of in-line separation has taken the confidence of the synthesis community one step ahead [21,34–35]. In parallel to this, in-line analytical techniques have also been used for on-line measurement and characterization [36–38]. Multistep flow synthesis is a significant milestone in practice of organic synthesis. In the recent time, there has been a visible surge in the number of publications on multistep flow synthesis with specific target molecules [26,39]. Table 2 shows a few drugs which are synthesized using multistep flow synthesis. Multistep flow synthesis approach has the capability of replacing the conventional synthesis methods. It involves many unit operations also made to operate continuously to truly harness the benefits of flow chemistry which is not an easy task.

**Table 2:** A few important drug molecules synthesized in multistep continuous flow.

molecules and reaction/separation steps	end product	remarks

olanzapine (Zyprexa) [11] • 4 reaction steps • 2 separation steps	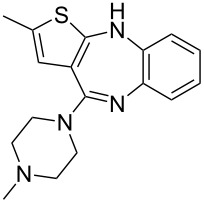	• antipsychotic drug • inductive heating was used • starting materials used: aryl iodide, aminothiazole Pd_2_dba_3_, xantphos, Bu_4_NOAc, Et_3_SiH, HCl, piperazine
tamoxifen [12] • 5 reaction steps	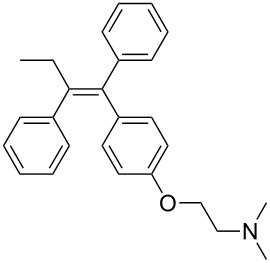	• breast cancer drug • telescope synthesis • moisture sensitive reagents were used • starting materials used: Weinreb amide, PhMgBr, aryl bromide, *n*-BuLi, aq HCl, TFAA, Et_3_N
amitriptyline [10] • 6 reaction steps	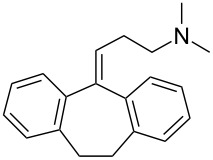	• antidepressant drug • moisture sensitive reagents were used • tube-in-tube reactor was used • inductive heating was used • starting materials used: benzyl bromide, *n*-BuLi, CO_2_, Grignard reagent, EtOH
rufinamide [40] • 3 reaction steps	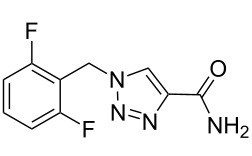	• anticonvulsant drug • telescope synthesis • copper tubing was used as reactor and catalyst • starting materials used: aryl bromide, NaN_3_, methyl propiolate, aq NH_3_
artemisinin [41] • 3 reaction steps • 4 separation steps	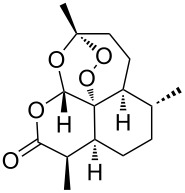	• antimalarial drug • the pressure was monitored to avoid unsafe backpressure due to clogging • starting materials used: dihydroartemisinic acid, TFA, toluene, O_2_, TMOF/TEOF/succinic anhydride
telemisartan [42] • 3 reaction steps	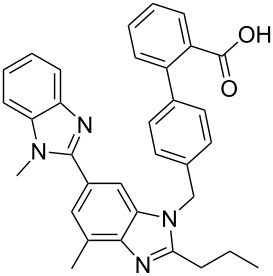	• hypertension drug • telescope synthesis • starting materials used: benzimidazole derivative, *t*-BuOK, bromide derivative, aq KOH, bromobenzimidazole
ibuprofen [43] • 3 reaction steps • 1 separation step	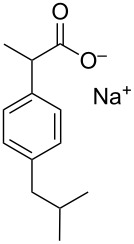	• nonsteroidal anti-inflammatory drug • three minutes residence time • starting materials used: isobutylbenzene, propionyl chloride, AlCl_3_, TMOF, ICl, NaOH, 2-mercaptoethanol
(*S*)-rolipram [7] • 4 reaction steps	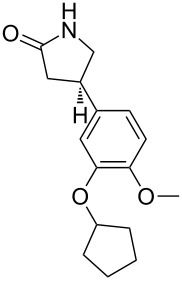	• anti-inflammatory drug and selective phosphodiesterase 4 (PDE4) inhibitor • heterogeneous catalysts • starting materials used: aldehyde derivative, nitromethane, malonate, Et_3_N, H_2_, water and *o*-xylene
(±)-pregabalin [44] • 3 reaction steps	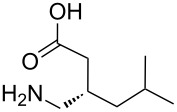	• used as a therapeutic agent for nervous system disorders such as epilepsy, anxiety disorder, and neuropathic pain • heterogeneous catalysts • starting materials used: isovaleraldehyde, methyl malonate, nitromethane, 1-PrOH, H_2_, HCl, NaOH

The utilization of the same approach for the synthesis of a wide range of products is very challenging since each product in the chemical synthesis involves different synthesis procedures, different conditions, different phases and different isolation protocols. However, the approaches adopted for several multistep flow synthesis still lack from seamless extrapolation to other synthesis platforms, including the non-availability of specific unit operation in continuous mode at the throughputs suitable for laboratory scale. Though, the multistep continuous flow synthesis approach is very promising for the synthesis of important chemicals having applications as medicinal drugs, agrochemicals, perfumery compounds etc., in general, the components/equipment in a flow synthesis platform are almost identical and this paves the way to think of developing a unified flow synthesis platform that can facilitate multistep synthesis involving a wider range of reactions over a varied range of conditions. Such a platform would help to reduce the time in planning of experimental set-ups for individual reaction(s) or sequences and will also help to do a seamless integration of experimental conditions with smaller laboratory footprint. In addition to the most obvious purpose of having such a platform that will facilitate the synthesis of any molecule including several intermediate stages, it will help in terms of the following:

1. **End-to-end synthesis:** Total synthesis of various molecules involving multiple chemical transformations (homogeneous or reactions involving multiple phases) at various optimal conditions including work-up/purification in continuous mode.
2. **Screening:** Rapid screening of operating conditions and development of a library of molecules from similar initial substrates.
3. **Convenience:** Selection of the specific parts of the set-up for a given synthesis step or for selecting a sequence of reaction steps reduces the time to disassemble and reassemble the set-up for different products. So, operating the set-up and deciding the parameters for each step becomes convenient using a unified platform.
4. **Modularity in true sense:** Making the reaction platform having plug-and-play approach would make it modular in true sense.
5. **Adaptability:** Having components with multiple functions will reduce the overall number of equipment/instruments on the synthesis platform.
6. **Automation:** Reduced human intervention facilitated by in-line measurements, automated optimization programs and continuous operation for a controlled set of conditions will be the unique features that will make such platforms attractive and efficient.
7. **Reproducibility:** Development of individual reaction steps and their optimization at various locations of an organization can become reproducible upon integration through such platforms.

While the concept of a unified synthesis platform looks fascinating and useful to reach the targets like ‘Dial-a-molecule’ [45], in reality, it can be very challenging. Some of the challenges are as follows:

1. **A varied range of conditions:** A multistep synthesis platform developed for one target molecule cannot always be utilized for different products since each product either requires different chemistry or a different set of unit operations or unit operation sequences. In some cases, synthesis and chemistries can be very different such that totally different set of flow reactors (including material of construction) and operating conditions has to be employed, e.g., flow chemistry literature shows the use of a wide variety of flow reactors, e.g., tube-in-tube gas permeable membrane reactors [46–48], high-pressure reactors utilizing back pressure regulators [49–51], reactors with different heating and cooling modes (e.g., inductive heating [11,52], microwave [53–55] etc.) and many more, also very special reactors [56] with other difficulties that need to be taken care. Also reactions are varied in terms of conditions such as the utilization of novel process windows [57–59] where high temperature and pressure is utilized which needs special attention in terms of safety and other criteria compared to the reactions requiring ambient conditions and low to moderate temperatures.
2. **Matching of time scales:** Residence time associated with a specific operating condition in each reactor and in a separation protocol (i.e., unit operation) in sequence has to be matched properly to get the desired final product which needs to be optimized every time if the throughput in the start or anywhere else gets changed in the sequence. This is very important for synthesis steps where downstream processing is also in sequence. Usually time scales for work-up procedures like extraction, crystallization, solvent switch etc. are longer compared to the main reaction and for any particular reaction in sequence the time scale for all other steps has to be either fixed or it gets fixed based on the initial step. One option is to have more pumps and collect the reaction mass at some point to change the flow rate for matching of time scale [7–9,20]. However, such an arrangement is complex and makes it very difficult to vary for each new scenario which requires special skill set or modification in chemical step.
3. **Suitability of control structure and sensitivity:** A multistep flow synthesis approach possesses challenges in terms of controls where a slight change anywhere in the process sequence can hamper the product output or will require very different kind of control strategies in the subsequent steps. For example, the reaction can be sensitive towards mixing, mass transfer/flow regime, temperature, etc. Slight variation in pump flow rate or coolant flow rate/temperature can change the relative time scales of the process affecting its selectivity. For such cases, the control system should quickly bring the process to steady state to maintain the desired selectivity. Shukla and Kulkarni have reported a control structure for a few synthetically important drug molecules and discussed challenges involved in developing such a control process [60].
4. **Monitoring:** Utilization of in-line analysis techniques and constant monitoring of the product also requires specialized equipment to be used and relative ‘analysis time’ in the whole process sequence is much greater compared to the reaction time. During utilization of such in-line techniques like HPLC, UV and IR etc. where analysis time is greater than reaction, provision has to be provided for intermittent sampling to monitor the reaction progress. In those instances it is the analysis time that dictates the control structure and parameters to be varied in case of any disturbance at/during any stage of operation [38,61].
5. **Optimization:** In continuation to the first point above, since every reaction step would have a different set of optimal conditions, the availability of a varied range of utility (i.e., the heating or cooling systems) and their suitability for integration on a single platform would be challenging for configuring the entire platform. Moreover, even after realizing such a platform, optimal conditions for each step would be different this might need significant reconfiguration for making a real ‘plug-and-play’ kind of system. This means that the unified synthesis platform should have a number of utility variations as low as possible.
6. **Compatibility:** The material of construction or make of the process components may not be always suitable for a given set of reactants/products/solvents/byproducts. Even the change in the sequence should be adaptable such a system can be very expensive as well.
7. **Skills:** With the advent of many flow synthesis tools available in the market much of the above issues may be taken care of. However, the automation in multistep synthesis needs careful selection. In general, setting-up of a multistep flow synthesis platform is very time consuming and needs multidisciplinary skills or a bigger team as it gets reflected in a few excellent works from the literature [8–9,20,62].

### Motivation

In view of the above introduction, in rest of this manuscript, we have explored the feasibility of having a unified multistep flow synthesis platform which can help to do almost any flow synthesis. Such a platform, if developed would resolve most of the above-stated challenges and will reduce the time and other resources whenever new chemistry has to be developed in continuous flow manner. The proposed platform will contain all the necessary components of a multistep synthesis unit that will be sufficient to perform a number of chemical syntheses with wide variation in synthesis steps. With the developed platform it will be very easy to do a screening of different chemistries and save a lot of time for beginner chemists in terms of locating and assembling the setup. The proposed approaches are more as a guideline and will need elaborate engineering analysis before actually building them. However, we have also given specific recommendations in that direction. Before presenting and evaluating various approaches for building a unified multistep synthesis platform in Table 3 we have given definitions of a few terms used throughout the manuscript and their relevance.

**Table 3:** Definition of the specific terms used in the article.

terms as used in this article	meaning/relevance

1. reactor	the section of the platform used for carrying out reactions. Usually, reactors are followed by separators (for extraction, distillation, chromatographic separation, crystallization, etc.).
2. instrument	wireless or cabled electronic unit that interfaces with the reactor and separator to facilitate monitoring and/or measurement and/or control.
3. equipment	an electronic unit that facilitates dosing of gas, liquid and solid.
4. component	connecting joints between reactor(s), instruments and equipment. These will include fittings, connectors, valves, etc.
5. module	an assembly of all the above segments to facilitate flow synthesis along with monitoring and control (1–4).
6. variables and parameter	set of conditions (set points or variables) that are used for optimizing a specific reaction section or the entire sequence of reactions.
7. stage	individual unit operations (viz. pre-heating, mixing, reaction, quenching, separation, etc.).
8. number of steps	number of reactions (chemical transformations) in a sequence to obtain the final product.
9. synthesis sequence	a sequence of reactions and unit operations (stages) in the synthesis path for the specific final product.

### Design complexity

A general flow chemistry setup requires some basic equipment like pumps, reactors (usually a flow reactor tube of required length and diameter or a microchannel reactor having various geometries or a static mixer) or a continuous stirred tank reactor or a fixed bed reactor or other intensified process equipment viz. spinning disc reactor, impinging jet reactor etc.) and a thermostat which will maintain the reaction temperature and components viz. valves, measurement devices and so on. As mentioned earlier, a list of various terms used in this article is given in Table 3. The functionality and nature of the setup can change with the chemistry under investigation and the experience of an individual involved in handling simple to complex synthesis containing a large number of stages and components. This demands more attention to address a few important aspects of such a unified synthesis platform.

**Component selection:** Component selection is the most important task for designing any synthesis set-up that targets a specific product. For a typical multistep flow synthesis involving several reaction stages, the system will require several components, reactors, and equipment. One can definitely identify some class of reaction where the same kind and number of components can be utilized but a slight change in synthesis route/chemistry will require a new component to be added extra or for the same component the suitable material of construction might be different than before. This can lead to a bulky system having a complex flow path.

**Choice of parameters:** Choice of a range of operating conditions/parameters is a very crucial aspect while designing a unified synthesis platform. In a multistep synthesis route, each stage will have its own set of operating conditions for getting the optimum yield. A set of reactors and components designed for a specific reaction would require optimization in terms of operating conditions to match the throughput or residence time when used for another reaction. Moreover, once the system or synthesis platform is built, any minor variation needed at one stage due to possible variation in the purity of reactants will require manipulation at each stage in the sequence.

**Number of steps:** The number of reaction steps and subsequent downstream processing for the synthesis of any final drug molecule or an agrochemical is usually different. Therefore the components needed for a specific synthesis protocol will also vary. Thus a unified multistep flow synthesis platform may not be adequate and cannot be complete for the synthesis of any and every molecule. For example, a few synthesis steps need very specific type of equipment (viz. ozonolysis), which is not needed in every routine synthesis.

**Sequencing of components:** For a unified synthesis platform to become adaptive to any kind of reaction sequence (reaction followed by separation and purification) is one of the most important design challenges. As the component in a platform would be fixed, for every synthesis either some components must be bypassed or connected in a loop, which would increase the dead volume in the overall system. This would enhance the residence time, demand more safety features and also need more inventory. A larger dead volume has its own challenges.

**Control strategy:** Devising a control strategy for a unified synthesis platform itself will be the most complex task. The complexity will originate from the varied control structures needed for individual synthesis sequence. For every reaction sequence verification of the sensitivity bounds on the specific control, strategy has to be developed for optimum performance of the setup.

**Scale of operation:** Throughput for any targeted molecule may vary based on the user requirement. Choosing a component to be operated in up-scaling and down-scaling mode at several throughputs with a wide range of operating conditions is very difficult. More than the effect of residence time, the hydrodynamics for the same reactor would vary depending upon the throughput and will affect the performance severely. In such a case, the plug-and-play mode might work provided the change of component is limited and absolutely necessary.

**Troubleshooting:** As a unified platform will involve lots of components for a chosen multistep synthesis flow path, the standard protocols for start-up, operation and shut-down will vary depending upon the reaction sequence. Thus, the interlocks and control structure should be updated accordingly. For example, among the presently available automated flow synthesis platforms, the limitation always comes from non-availability of troubleshooting protocols.

**Simultaneous use for synthesis of different molecules:** Having a unified platform will serve the purpose only if all the units on the platform are utilized all the time which may not be the case always. Utilizing all the components simultaneously for different synthesis sequence will need isolation of one flow path from the other and since the whole system is integrated, this will introduce complex operational challenges.

**Utility optimization:** The operating conditions for individual reactions in a sequence are usually different and the reaction temperature can vary from −78 °C < *T* < 200 °C. In such a situation, it cannot be a viable option to have a different utility for individual reaction steps.

The above mentioned specific points need to be taken into account while planning for a unified synthesis platform for flow synthesis. Thus, depending upon the set of targeted molecules or functional group transformations it is possible to propose several design/assembly options. In rest of this article, we bring out a few different ways in which it would be possible to design a unified flow synthesis platform. A few case studies from the literature on multistep flow synthesis of very specific drug molecules are used to explore and evaluate the design approach for building a single synthesis platform that can help produce all of those drug molecules, each having a very different synthesis route.

### How do we use it for drug synthesis?

The proposed options of a unified synthesis platform will serve as a convenient tool at lab scale. Many new chemistries that are parts of a multistep flow synthesis route are to be performed with slight changes in the component/layout. The platform will serve as a single destination for the multistep flow synthesis whenever a reaction has to be optimized or new screening has to be done. It is expected that with slight modifications, a user will be able to ‘choose’ a multistep synthesis flow path in the unified platform.

### Approach

For designing such a system we have analyzed the literature on multistep flow synthesis of API’s through complex chemistry. We have shortlisted the papers which contained different equipment’s used in the pharmaceutical manufacturing to cover most of the functional groups which can be organized on the single platform and can be utilized for a number of chemical syntheses.

After identification of specific molecules to be used for developing a unified synthesis platform we have identified the number of components associated with a synthesis and then optimized the number of component which will be sufficient to do all the identified reactions. Once the components were chosen the optimal sequencing which will be efficient to do the reactions without much difficulty has been developed and sequencing was done. In order to evaluate the feasibility of the above concept, we have considered different multistep syntheses as case studies. Number of steps, starting material and other conditions are listed in Table 4.

**Table 4:** Multistep synthesis case studies selected for the article.

**multistep synthesis of (*S*)-rolipram** [7] 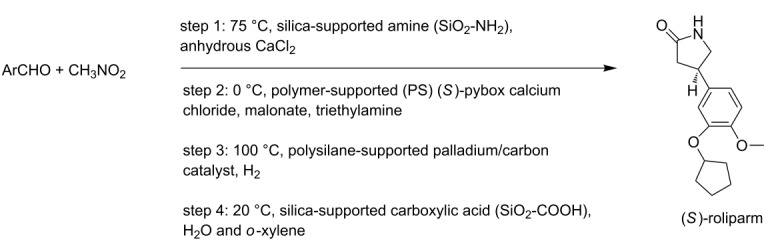

**multistep synthesis of ribociclib** [63] 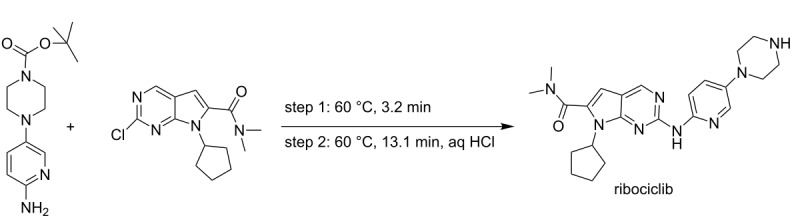

**multistep synthesis of prexasertib monolactate monohydrate** [8] 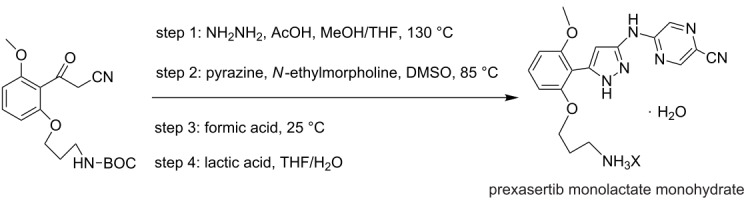

**multistep synthesis of lidocaine hydrochloride** [9] 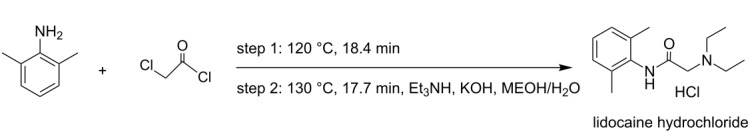

**multistep synthesis of fluoxetine hydrochloride** [9] 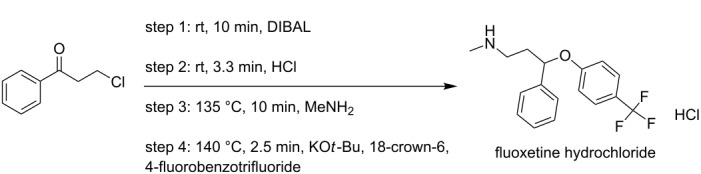

**multistep synthesis of diphenhydramine hydrochloride** [9] 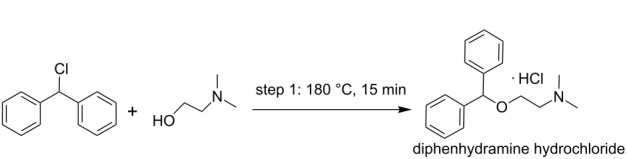

**multistep synthesis of diazepam** [9] 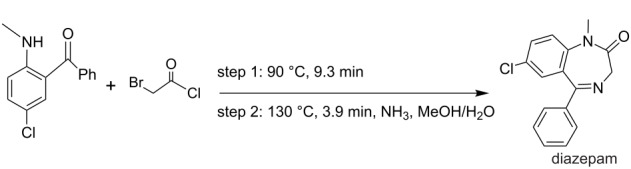

**Figure 1:**
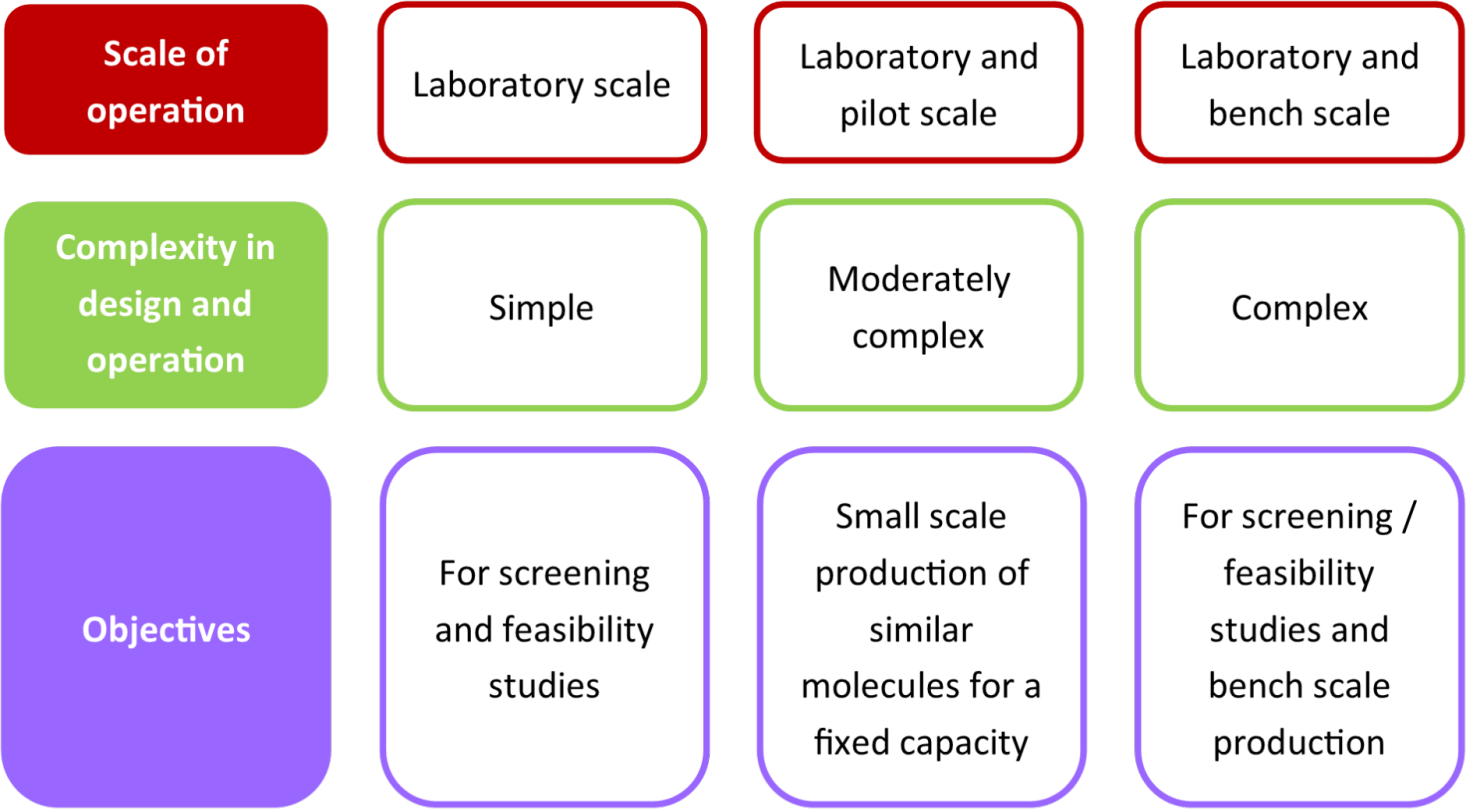
Key features of different approaches for unified multistep synthesis platform.

For these few cases we have evaluated three different approaches that can be used for developing a single synthesis platform. Every approach is based on a different logic of making a unified multistep flow synthesis platform. Figure 1 shows a comparison between the different approaches.

#### Approach 1: Unimolecular synthesis (one at a time)

**Figure 2:**
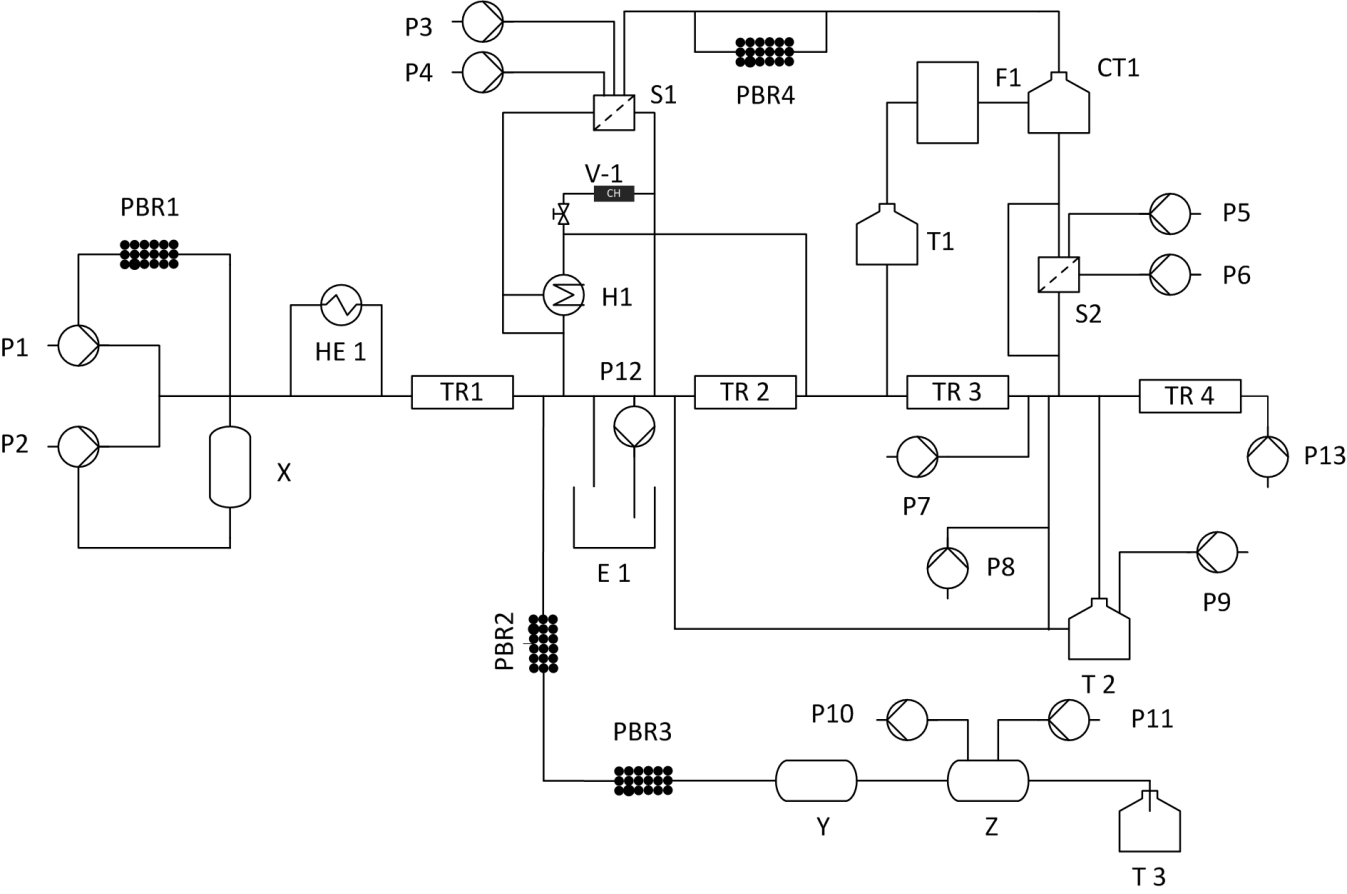
Schematic representation of a unified platform for the flow synthesis (P1–P14 pumps, PBR packed bed reactor, HE1 heat exchanger, H1 heater, S1 and S2 separator, E1 extractor, TR1–TR4 tubular reactor, CH charcoal, CT1 crystallization tank, T1–T3 tanks, F1 filtration).

The first approach towards development of a unified flow synthesis platform mainly aims at minimizing the number of components and to perform the reactions in a single system without much change of components (Figure 2). Here the components are fixed on one platform and the synthesis of a specific compound is carried out by choosing the path which is required for the reaction and other paths are blocked by using automated valves. This approach is good for relatively simple reactions and for some complicated reactions the number of components increases that lead to a large number of connections and a complex control structure. Table 5 shows the path for the synthesis of different products based on approach 1, Table 6 shows a list of components required for the synthesis of the above products using approach 1.

**Table 5:** Conventional path for the synthesis of different intermediates based on approach 1.

intermediate	multistep synthesis flow path

prexasertib monolactate monohydrate	P1+P2→HE1→TR1→E1→TR2→TR3→RE1→T2→TR4→F1→T1
aliskiren hemifumatate	P1+P2→R1→S1→S2→TR4→S1→PBC→C1→S2→T2
diphenhydramine hydrochloride	P1+P2→R1→H1→BPR→CH→S1
lidocaine hydrochloride	P1+P2→R1→R2→BPR→CH→S1
diazepam	P1+P2→R1→R2→BPR→CH→S1
fluoxetine hydrochloride	P1+P2→R1→R2→S1→S2→R3→S1→H1→R2→T1
ricociclib	P1+P2→R1→R2→S1→R4→T1
rolipram	P1+P2→PBR1→X→TR1→PBR2→PBR3→Y→Z→T3

**Table 6:** Components required for the synthesis of the above API’s [pumps (P), reactor (R), heat exchanger (HEx), heater (H), back pressure regulator (BPR), packed/fixed bed reactor (PBR/FBR), separator (S), charcoal adsorption cartridge (CA), liquid–liquid extractor (LLEx)]

name of API’s	P	R	HEx	H	BPR	PBR/FBR	S	CA	LLEx

diphenhydramine hydrochloride	4	1	–	1	1	–	1	1	–
lidocaine hydrochloride	5	2	–	–	1	1	1	–	–
diazepam	4	2	–	–	1	1	1	1	–
fluoxetine hydrochloride	11	4	–	1	4	–	4	–	–
aliskiren hemifumarate	14	2	–	–	–	1	5	–	–
ricociclib	4	2	–	–	–	–	2	–	2
rolipram	7	1				5			
prexasertib monolactate monohydrate	20	3	1		–	–	1	–	2

Figure 2 involves the platform for the synthesis of API’s listed in Table 5. One example has chosen from Table 5 to explain approach 1. The description for the synthesis of prexasertib monolactate monohydrates based on approach 1 in Figure 2 is explained as follows: The synthesis of Prexasertib monolactate monohydrates involves four steps a) condensation b) aromatic nucleophilic substitution reaction, c) deprotection and d) formation of lactate salt. Details of the same are given below:

- **Condensation:** Condensation takes place in a first reactor TR1 between the nitrile and hydrazine at high temperature and under pressure. Here, the nitrile was dissolved in a THF and hydrazine was dissolved in a mixture of solvents such as methanol, acetone, and water. The nitrile was pumped using pump P1 and hydrazine was pumped through P2 into the tubular reactor TR1 maintained at a temperature of 130 °C at residence time of 60 minutes to obtain the pyrazole. The impurities of the pyrazole were removed by passing it to the continuous countercurrent extraction E1. Here a solvent exchange process takes place between toluene and water. The pyrazole was then concentrated using automated rotary evaporator RE1. The concentrated product was diluted with DMSO using pump P13.
- **Aromatic nucleophilic substitution:** The nucleophilic substitution reaction takes place between the pyrazole and *N*-ethylmorpholine. Pyrazole of step 1 in the extractor was pumped through P13 and *N*-ethylmorpholine through P3 into the reactor TR2 to form the arylated product of the pyrazole. Here the reactor was maintained at a temperature of 70–100 °C for 1–3 hours. The product was crystallized in CT1 with the anti-solvent methanol pumped through P4 into the crystallization tank. The crystallized product was filtered and separated in S2.
- **Deprotection:** The second-stage product from the separator enters into the tubular reactor TR3 at a temperature of 20–40 °C with a residence time of 4 hours. Into this reactor nitrogen gas was pumped through peristaltic pump P7 and formic acid using pump P8. In TR3 gas–liquid reaction takes place.
- **Formation of the lactate salt:** In step four, lactic acid was pumped through pump P3 to form the final lactate salt of the product. Here the excess of formic acid and lactic acid was removed by the rotary evaporator RE1, then passes through TR4 into the crystallization tank CT1. The solid product formed was filtered in F1 and stored in a tank T1.

**Challenges in performing multiple reactions in a single platform as given above:** The number of valves needed to select the desired set of equipment is much higher. The reactions which take place only in a packed bed reactor and do not involve a separator, filter, crystallizer, etc. The path required for the synthesis is the same as that of synthesizing it individually so that the number of components required will remain unchanged and it is the same as that of an individual synthesis.

#### Approach 2: multimolecular operation (more than 1 molecule at a time)

This approach consists of identifying and optimizing a minimum number of components for performing flow synthesis of different molecules. The developed platform will contain all the necessary components for synthesis (flow reactors, packed columns etc.) to the downstream processing (extractor, separator, crystallizers, dilution tank etc.). Some of these components can be used for different chemistries just by changing the flow rates or the operating conditions specific to the chemistry. The components will be arranged on a platform where the order of arrangement can be varied in terms of processing needed for chemical synthesis just by connecting the components via tubes. The designed platform will be provided with some accessories which will include at least one component of all types on the platform (of different or same volume, or suitable to the different operational parameters) with an exactly same dimension which will make replacement of a component easy in case of failure or whenever needed. This platform will be a plug-and-play kind of system where the user will just have to choose the specific order of the component arrangement and to select the operating parameters before starting any experiment. The platform can be used for a specific synthesis step optimization or for performing an optimized multistep synthesis. The plug-and-play approach makes it very useful in the sense that if some or any component on the platform is not being utilized for any synthesis that component can be removed and used for another purpose or simultaneous synthesis of different molecules can be done using components which are not being utilized for the ongoing synthesis. The components like dilution tanks, crystallization tanks, and gravity based liquid–liquid separators can serve different purposes if planned properly before the experiment so that the same component can be used interchangeably with different chemistries reducing the need for different components still further.

**Figure 3:**
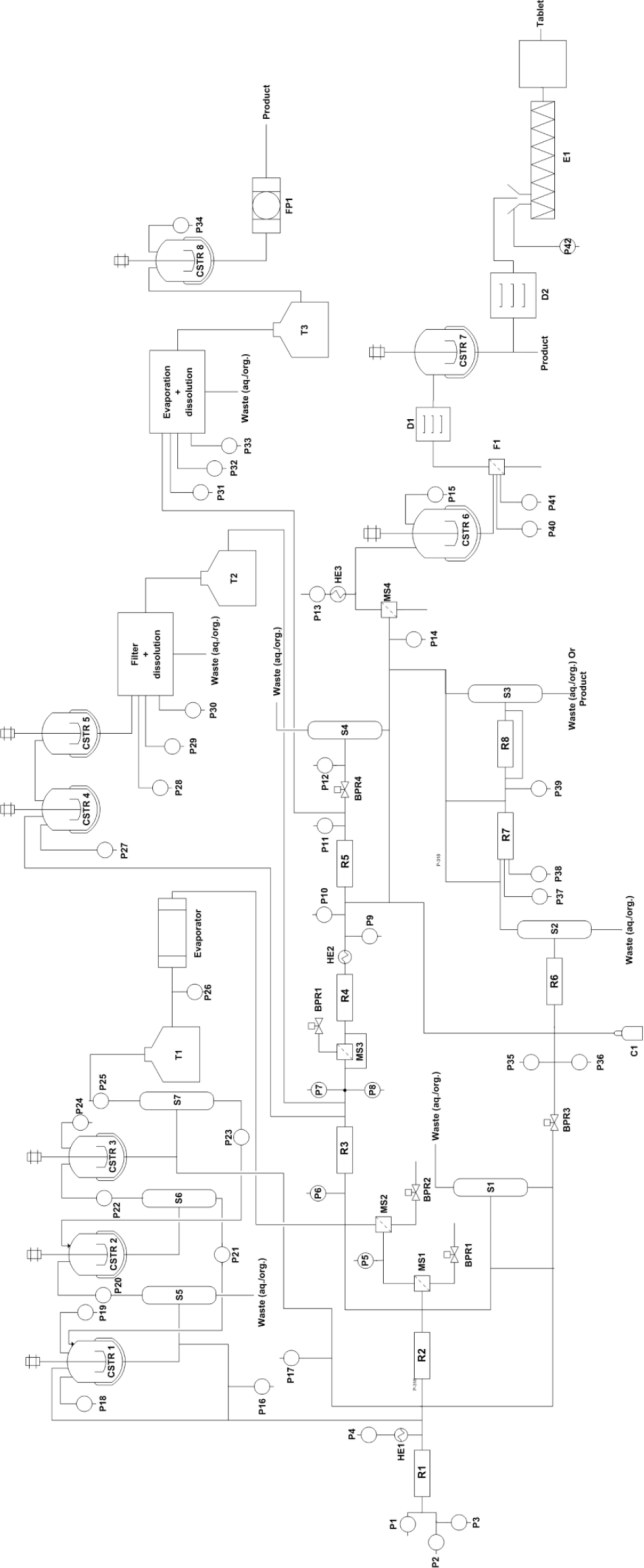
Layout of a unified synthesis platform (including all the component) for multiple drug molecules (approach 2) R – coil reactor/packed bed reactor/scavenger, P – pump, HE – heat exchanger, CSTR – stirred tank reactor/crystallizer/dilution tank, T – storage tank, F – filter, S – gravity-based separator, D – dryer, FP – filter press, MS – membrane separator, E – extruder, BPR – back pressure regulator.

**Figure 4:**
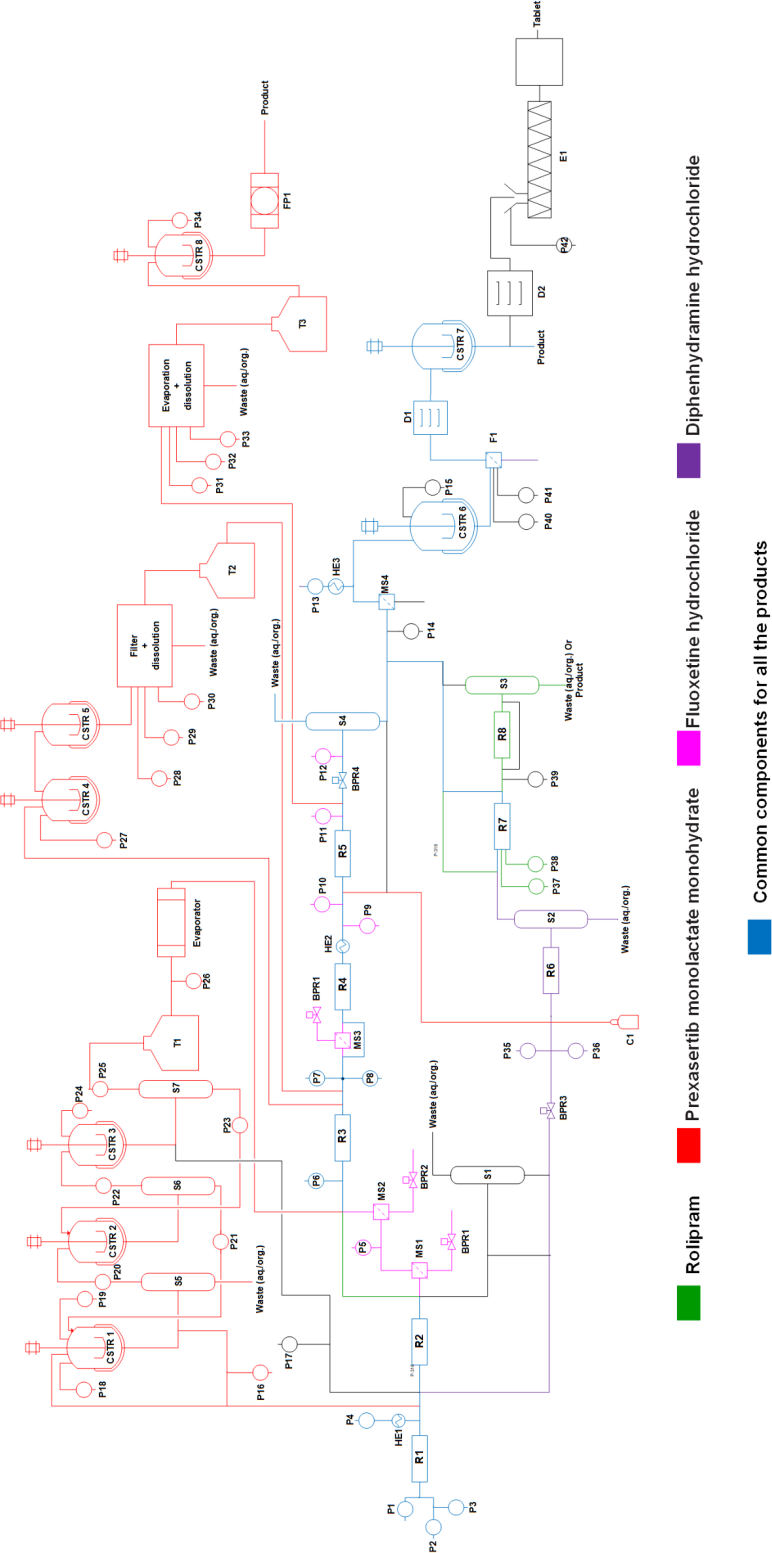
Layout for synthesis of 4 molecules on a single platform (approach 2).

Figure 3 shows the unified platform based on the approach 2 which contains the optimum component based details extracted from the literature of selected case studies. The sequence of components was arranged according to the described setup in the case studies selected. Figure 4 depicts 4 processes in one chart and the components in blue color are the common components, which will take part in the synthesis of any or every molecule chosen from the case studies. That reduces the quantity of the same kind of components by 4 times. The number of components for each unit operation is quite large, however, that helps to carry out the synthesis of all the identified products in the chart.

**Scheme 1:**
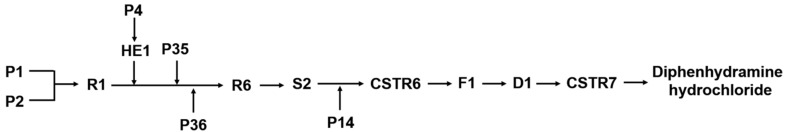
The overall process for the synthesis of diphenhydramine hydrochloride.

To have a view of the platform as in approach 2 one example of diphenhydramine hydrochloride is covered here from the case studies, where two reactants 2-dimethylaminoethanol and neat chlorodiphenylmethan is being pumped from P1 and P2 to reactor R1 where it is getting heated at a temperature of 180 °C at a pressure of 1.7 MPa. The molten salt which comes out of reactor R1 is then treated with aqueous NaOH through pump P4 which is heated to 140 °C through HE1. In-line extraction and purification happen in packed bed column reactor R6 by water and hexane which are pumped through pump P35 and P36. The resulting biphasic solution passes through gravity operated liquid–liquid separator S2 with automatic level control. In the downstream section the API was precipitated with HCL through pump P14 and the precipitate is dissolved in ethanol and crystallized in CSTR6 maintaining temperature at 5 °C. After the crystals are being filtered through F1 and dried in D1, the final product was dissolved in water in CSTR7. The final product diphenhydramine hydrochloride is collected in the form of a solution. The overall process follows the path as shown in the sequence of Scheme 1.

With this approach, it is very easy to reduce the number of components significantly to perform a number of different chemical steps of varying nature (except very different chemistries where very specific equipment is required). The platform developed using this approach will have the following key features:

1. **Useful for a limited number of molecules:** This approach will be very useful if a similar set of chemical transformation is to be performed which will reduce the number of components significantly, however, the approach discussed above is not unique since everyone can come up with an optimum number of components based on the chemistries involved and the level of expertise.

2. **Volume of each component:** Choosing the right component volume plays a very critical role here since that is going to fix the residence time and the overall throughput. For the same synthesis route, the volume of a component will vary if the throughput is going to increase or decrease. It becomes very important before designing such a platform to define the scale of operation and the type of chemistry that will be used since much of the selection criteria will depend on the aforesaid two parameters.

3. **A number of components:** As components can be interchanged and reused, defining the number of components is not critical but one has to take this into account since this will depict the overall costs of building such a platform. Though one can have a large number of accessories, adding each one on the synthesis platform will increase the cost.

4. **Connection for components:** Connecting the components in proper sequence is required for success in any multistep flow synthesis including work-up. Making a connection before and after each operation will add an extra volume to the existing process volume, which needs to be taken care off. In this approach, the connection is not fixed rather the plug-and-play kind of approach can bring the components close to each other reducing the need for intermediate heating/cooling or the requirement of an additional utility to maintain the reaction temperature in the tubes.

5. **Instrumentation:** Here, we have not explicitly considered any instrumentation (other than in-line analysis or measurements for monitoring a given reaction/purification) but that can be added at the specific steps wherever needed.

6. **Utility:** At this point of time it is assumed that for each reaction step the heating or cooling arrangement (also referred as ‘utility’ in the chemical process engineering and plant operation) is arranged individually.

#### Approach 3: a cybernetic approach

**Figure 5:**
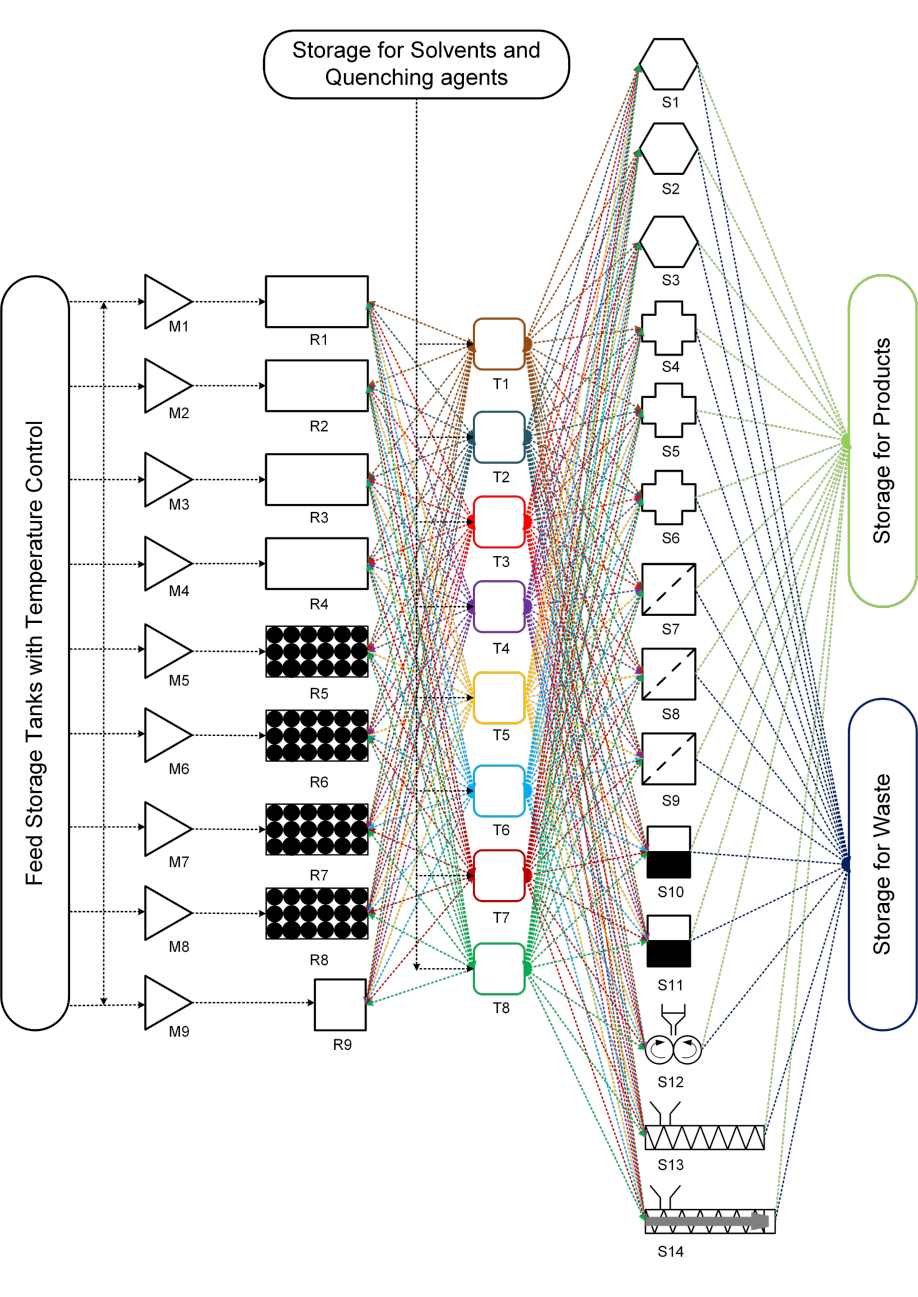
Approach 3 for a unified platform for multistep synthesis. M1–M9 = mixers, R1–R4 = tubular reactors, R5–R8 = packed bed reactor, R9 = stirred tank reactor, T1–T8 = Intermediate storage tanks, S1–S3 = adsorption columns, S4–S6 = extraction columns/gravity-based separator, S7–S9 = membrane separator/Filter, S10–S11 = evaporator, S12 = rotary drum dryer, S13 = vacuum screw dryer, S14 = extruder.

The third approach can be based on the need for a versatile and extremely flexible system. Figure 5 shows the concept of a unified platform (Approach 3) for multistep synthesis in a continuous flow. The platform can have three basic modules which are interconnected.

The first, reactor module includes different reactors types that are commonly used in the synthesis of APIs viz. tubular reactor (R1–R4), packed bed reactor (R5–R8) and stirred tank reactor (R9). The reactors are equipped with a jacket for maintaining the reaction temperatures. Additionally, multiple temperature zones can also be provided if required. The reactor module also includes mixers (M1–M9) that are commonly used in flow chemistry [64–65]. The continuous flow reactor can also be equipped with in-line static mixing elements [63].

The second module includes the intermediate storage tanks (T1–T8) with an agitator and a jacket for maintaining the temperature. The intermediate storage tanks can be used for multiple purposes viz. preheating/precooling any reaction intermediate, mixing reagents, quenching the reaction, dilution, crystallization, reaction and can be operated in batch or continuous mode (CSTRs). Preheating and precooling are essential for getting reproducible and reliable experimental data.

The third and final module includes separators viz. membrane separators/filters, scavengers or adsorption column (packed column), extractors/gravity separators, dryers, extruders, etc. These three modules can be fixed in a 3D space on a skid. However, the tubings, valves and back pressure regulators need not be fixed and can remain connected to individual module units as per process requirements. Avoiding the tubing will add more flexibility to the unified platform similar to pipeless plants [66]. The entire platform has separate tanks for storing the feed, product, solvent/buffer solution for extraction and waste collection. The feed storage tanks will be equipped with temperature control for preheating or precooling of any reagent before mixing. Moreover, the unified platform can be integrated into any commercial separation and analytical system. This approach is analogous to cybernetics [67].

Table 7 shows the process components required and the sequence of unit operations for producing various pharmaceutical products using approach 3. These unit modules can be connected in the desired sequence by connecting the tubing. Additionally, valves and back pressure regulators can be used whenever required. The unit operations which are not required for the process under consideration will not be connected. This approach allows connecting any unit operation in any desired sequence making it a unified platform for multistep synthesis. Such a platform can be integrated with chromatography purification systems, in-line analytical instruments, a mold for tablet making and various commercial instruments.

**Table 7:** Sequence of unit operations for various pharmaceutical products by approach 3.

reference	product	reactors/equipment/ components (number)	sequence of unit operations as per approach 3 (see Figure 5)

Tsubogo et al. [7]	(*R*)- and (*S*)-rolipram	• packed bed reactors (4) • adsorption columns (3)	M5→R5→T5→S1→M6→R6→T6→M7→ R7→T7→S2→T8→S3→R8
Pellegatti et al. [63]	ribociclib	• flow reactors (2) • stirred tank reactor (1)	M1→R1→M2→R2→S4→T1→M3→R3→ S5→R9
Cole et al. [8]	prexasertib monolactate monohydrate	• flow reactors (4)	M1→R1→T1→S4→T2→S5→T3→S6→T4 →S10→M2→R2→T5→T6→S7→S8→T7
Adamo et al. [9]	fluoxetine hydrochloride	• flow reactors (4)	M1→R1→T1→S7→T2→S8→R2→T3→ S10→A1→T4→R3→T5→S4→downstream
	diazepam	• flow reactors (2)	M1→R1→M2→R2→T1→R5→S4→S1→ T2→S5→ downstream
	lidocaine hydrochloride	• flow reactors (2)	M1→R1→N2→R2→T1→R5→ S4→downstream
	diphenhydramine hydrochloride	• flow reactor (1)	M1→R1→T1→R5→S4→S1→downstream
Mascia et al. [20]	aliskiren hemifumarate	• flow reactors (2) • crystallizers + tanks (6)	M1→R1→T1→S4→T2→T3→S7→T4→M2 →R2→T5→S5→S8→S1→T6→T7→S9→ T8→S12→S13→S14→moulding machine

As suggested, a priory information should be known regarding kinetics of various processes (reaction/drying/crystallization/adsorption/desorption), solubility data (extraction/crystallization), etc. This approach is useful for testing proof of the concept for a continuous process of various drugs which are in clinical trials. However, this approach may not be feasible for pilot or production scale as the scale of operation is different and reactors and separators should be designed accordingly. The ideal use of this platform is to evaluate the possibility of the synthesis concept of various processes along with automation having a variety of unit operations and operating conditions and collect useful data for further plant design or for using it for a specific period of time to meet the production needs and then switch to another molecule, making it a flexible production platform. Eventually, at the pilot or production scale, it will be analogous to approach 2. Key features of this approach can be given as follows: (i) truly unified multistep flow synthesis platform, (ii) intermediate tanks can be used for preheating/precooling, isolating different pressure zones and intermediate storage, (iii) the system will have all the necessary components like back pressure regulator, check valve, control valve, temperature and pressure sensors, etc., (iv) the stirred tank reactor can be used for the reaction and also for crystallization, (v) the reactor jacket can have multiple temperature zones to offer more flexibility, (vi) the fixed bed/packed columns can be used as reactors as well as scavenging columns depending on the requirement or even as a mixer if the packing is inert.

While such a unified platform would offer enormous flexibility in operation, it would be challenging to develop such a platform. A few challenges can be given as follows: (i) too many connections, (ii) arrangement of various components in 3D space is critical, (iii) needs very complex control strategy, (iv) minimizing the pipeline length during component assembly is challenging to optimize the residence time variation and will handle more chemicals than conventional systems, (v) relatively large amount feed material will be required when compared (to compensate dead volume) to a single dedicated experimental setup and (vi) automation will be complex as well as expensive.

#### Simultaneous synthesis of (*S*)-rolipram and ribociclib by approach 3

The aldehyde and nitromethane are dissolved in toluene separately and kept in the feed storage tanks for preheating (see Figure 5). The reagents can be pumped with a suitable pump (viz., peristaltic pump, piston pump, diaphragm pump, etc.) into the mixer M5 and subsequently to reactor R5 which is packed with SiO_2_-NH_2_ and CaCl_2_. The intermediate nitroalkane obtained is cooled to 0 °C in the intermediate storage tank T5. The reaction mixture can pass through separator S1 (adsorption column) which is packed with MS 4 Å to remove the byproduct water. A solution of malonate and triethylamine in toluene are precooled to 0 °C in feed storage tanks and pumped to mixer M6 where it is mixed with the nitroalkane stream. The reaction stream can then be passed through reactor R6 which is packed with polymer-supported (*S*)-pybox–calcium chloride and maintained at 0 °C. The reaction stream can be further passed to intermediate tank T6 where it can be preheated to 100 °C. The reaction stream containing Michael addition product is mixed with hydrogen gas (from H_2_ cylinder) in mixer M7. The resulting two-phase mixture can be passed to reactor R7 packed with Pd/DMPSi-C catalyst and maintained at 100 °C. The reaction stream can then be passed in intermediate tank T7 where unreacted hydrogen gas is vented and recycled and the liquid stream is preheated to 120 °C. The liquid stream then can pass through separator S2 (adsorption column) packed with Amberlyst-15 Dry to remove impurities. Water and *o*-xylene can be preheated and pumped from the feed storage tanks into intermediate storage tank T8 where it is mixed with the reaction mixture. The process stream can be further passed through separator S3 (adsorption column) packed with Celite. The reaction mixture can pass through reactor R8 packed with silica-supported carboxylic acid and maintained at 120 °C to obtain the product (*S*)-rolipram. In the above example, intermediate storage tanks T1–T4 can also be used instead of T5–T8 as every unit module (reactors, intermediate storage tanks, and separators) can be connected in any desired sequence by simple tube fittings. However, the choice of unit modules should be done on the basis of a lower tubing volume.

Chloropyrimidine and aminopyridine derivatives are dissolved in THF and can be preheated to 60 °C in the feed storage tank. LiHMDS solution in THF also can be preheated to 60 °C in the feed storage tank. Both the solutions can be pumped with suitable pumps in the mixer M1 and then through reactor R1 which is maintained at 60 °C. The product stream can be mixed with preheated HCl in mixer M2 and then passed through reactor R2 which is also maintained at 60 °C. The reaction mixture can then be passed to separator S4 (extractor) to separate the aqueous and organic phases. The organic waste can be collected in the waste storage and the aqueous phase is mixed with sodium hydroxide in intermediate storage tank T1 to quench the HCl. The reaction mixture can be mixed with THF in mixer M3 and passed through reactor R3. The process stream can be further passed to separator S5 (extractor) to separate the aqueous waste and organic phase. The organic phase can be further passed to reactor 9 (stirred tank) where it can be mixed with succinic acid for further batch crystallization to obtain the product ribociclib.

In this way, we can operate two synthetic processes simultaneously in the unified platform (approach 3). However, many unit modules still remain unused (viz., M4, R4, T2–T4, and S6–S14). Table 7 shows the unit module sequences for various products for approach 3.

## Conclusion

For the multistep flow synthesis approach, the next evolution is obviously towards a combination of automation, monitoring, screening, optimization, artificial intelligence and instrumentation. It has changed the conventional synthesis approaches through significant improvement in the product quality, efficiency, and smaller environmental foot print. Utilizing the benefits of multistep flow synthesis is not easy and it requires experienced professionals and ready-to-use tools for effortless integration of different synthesis stages. Developing the unified platform which will reduce the effort in setting up the experiments and integration of different component which will definitely help to speed up the overall process to truly harness the advantages of flow synthesis. Based on different objectives viz. reaction screening, library generation, bench/pilot scale synthesis for various molecules we have shown three approaches to make a unified multistep flow synthesis platform which can be made keeping the interest of individual or organization for future. These approaches show the unique and promising ways to make the unified platform to realize the concepts like dial a molecule. Realising the concept of a unified flow synthesis platform possesses some challenges but those can be taken care based on the need and planning beforehand. Once this platform is built it will act as ‘driverless car’ or a ‘robot chemist’ where an only instruction has to be given and platform will take care of the synthesis of the desired molecule based on the specific chosen flow path. The next level of such a platform can only go in the direction of self-regulated automatic 3D configurable synthesis platforms, just like an advanced version of ‘Transformers’. With growing machine intelligence, it is expected that the synthesis platforms would harness big data sets as a source of knowledge, artificial intelligence for decision-making abilities at various levels and self-optimization. Developing such a unified integrated multistep flow synthesis platform will be the new thing for organic synthesis to explore the unexplored chemistry.
